# Reliability of Miniaturized Transistors from the Perspective of Single-Defects

**DOI:** 10.3390/mi11080736

**Published:** 2020-07-29

**Authors:** Michael Waltl

**Affiliations:** Christian Doppler Laboratory for Single-Defect Spectroscopy (SDS) at the Institute for Microelectronics, TU Wien, Gusshausstrasse 27-29/E360, 1040 Vienna, Austria; waltl@iue.tuwien.ac.at

**Keywords:** device reliability, nanoscale transistor, bias temperature instabilities (BTI), defects, single-defect spectroscopy, non-radiative multiphonon (NMP) model, time-dependent defect spectroscopy

## Abstract

To analyze the reliability of semiconductor transistors, changes in the performance of the devices during operation are evaluated. A prominent effect altering the device behavior are the so called bias temperature instabilities (BTI), which emerge as a drift of the device threshold voltage over time. With ongoing miniaturization of the transistors towards a few tens of nanometer small devices the drift of the threshold voltage is observed to proceed in discrete steps. Quite interestingly, each of these steps correspond to charge capture or charge emission event of a certain defect in the atomic structure of the device. This observation paves the way for studying device reliability issues like BTI at the single-defect level. By considering single-defects the physical mechanism of charge trapping can be investigated very detailed. An in-depth understanding of the intricate charge trapping kinetics of the defects is essential for modeling of the device behavior and also for accurate estimation of the device lifetime amongst others. In this article the recent advancements in characterization, analysis and modeling of single-defects are reviewed.

## 1. Introduction

The complementary metal-oxide-semiconductor (CMOS) technology is the cornerstone of a vast number of integrated circuits, which are the building blocks of numerous electronic applications. Such circuits typically consist of a large number nMOS and pMOS transistors and their performance and geometry have been successively improved over the last decades. For instance, the width and length of the transistors have been reduced and the gate insulating layers have been thinned. Furthermore new device geometries such as FinFETs [1,2,3] and gate-all-around FETs [4,5,6,7] have been introduced. Notwithstanding this development, the reliable operation of the transistors at their nominal bias conditions is of utmost importance for all technologies. However, the most fundamental device parameters like the threshold voltage, the sub-threshold slope and the on-current, are affected by charge trapping at defects in the atomic structure of the devices. Such defects can be located at the interface between the insulator and substrate, but also inside the insulator and inside the semiconductor bulk material. In order to reduce the defect density of transistors post-oxidation annealing (POA) processes are applied during the fabrication process. The decisive importance of POA for improving the performance of transistors becomes even more obvious when Si and SiC based MOS devices are compared. While H${}_{2}$ annealing is regularly used within CMOS processes [8,9,10] similar POA steps could not lead to an improvement of the electron mobility in SiC devices [11]. However, by using NO or NH${}_{3}$ for POA, a considerable increase in carrier mobility can be observed for SiC MOS transistors [12,13].

Although a number of defects can become passivated using POA during fabrication, the interaction of high energetic carriers with atoms at the semiconductor/insulator interface during operation can break Si-H bonds and can lead to an electrically active dangling bond [14]. The bond rupture mechanism leading to the creation of interface states is typically referred to hot-carrier-degradation (HCD). In order to explain HCD in miniaturized devices the physical origin for HCD has been recently extended to cold carriers, where a series of collisions with low energetic particles can also lead to the creation of interfaces states [15]. Such an increase of dangling bonds at the interface can be observed as decrease of the device mobility, due to an increase of the interface scattering of carriers. The reduced mobility evolves as a reduction in the sub-threshold slope and can be for instance observed when IDVG measurements are performed [16,17], but can also be evaluated as the CV characteristics of the device alters [18].

**Figure 1:**
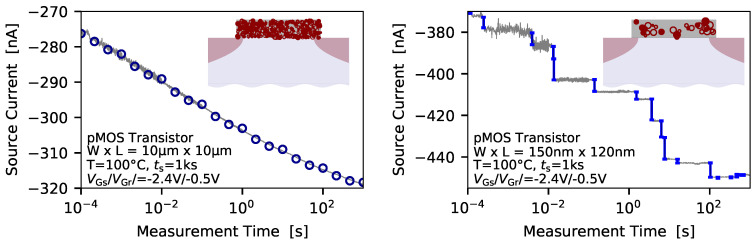
The main difference in the bias temperature instabilities (BTI) behavior of large-area and nanoscale devices is the number of defects contributing to the device behavior, and also the amplitude of impact of a single defect on the current flux through the device. (**left**) While in large-area devices a number of defects is responsible for a continuous drift of the drain-source current over time (**right**) the charge transitions of defects can be directly observed as discrete steps in the respective current signal recorded from nanoscale metal-oxide-semiconductor (MOS) transistors.

Another important reliability issue in miniaturized devices is the so called bias temperature instabilities (BTI) [19,20,21,22,23,24]. BTI typically manifest as a drift of the drain-source current over time when constant biases are applied to a transistor, and is studied up electric oxide fields of $E_{\mathrm{ox}}\leq 8MV/cm$. The physical origin of this phenomenon is charge trapping at defects which can be located at the semiconductor/oxide interface or directly in the oxide. The impact of BTI on the device behavior is mostly expressed in terms of an equivalent shift of the threshold voltage $\Delta V_{\mathrm{th}}$, which can for instance be calculated from the current measurement data using an initial IDVG characteristics of a device, when traditional measurement tools are used [25]. Alternatively, employing the fast-Vth method, where the gate bias is controlled by an operational amplifier in order to obtain a constant current flux through the device, allows for direct measurement of the $\Delta V_{\mathrm{th}}$ [26]. A typical temporal drift of the source current which can be measured when BTI is studied is shown in Figure 1 (left).

The inset indicates the number of defects affecting the device behavior. Quite interestingly, although the same physical mechanism are responsible for charge trapping in large-area and miniaturized devices, the picture of the drift of the device current is different for the scaled MOS transistors, see Figure 1 (right). While the source-current exhibits a continuous drift at large-area devices, charge trapping evolves in discrete steps of the device current, recorded at nanoscale MOS transistors. This is due to the fact that scaling of the devices on the one hand reduces the number of defects per device, but on the other hand the impact of a single defect on the overall device behavior gets considerably increased. Thus nanoscale devices inherently provide a zoom mechanism enabling to study charge trapping at the single-defect level.

The discrete steps in the current signal were first documented by Ralls et al. [27] and have since then been the basis for a number of investigations considering random telegraph noise (RTN) [28,29,30,31,32,33] aiming at the analysis of the physical origin of charge trapping. An significant advantage of evaluating RTN to conventional trapping analysis is that the charge capture and charge emission times can be extracted directly from single measurement traces. However, as only defects with a trap level close to the Fermi level of the conducting channel produce RTN signals tracing the bias and temperature dependence of the charge trapping kinetics of certain defect is limited to a very narrow bias and temperature range. To overcome this limitation and to enable a thorough study of the trapping behavior of a multitude of defects the time-dependent defect spectroscopy (TDDS) has been proposed [34,35]. The measurement sequences used for TDDS relies on the measure-stress-measure (MSM) scheme, which will be discussed in the following. Afterwards the TDDS is presented and finally charge trapping models and recent results from single defect studies are reviewed.

## 2. Measurement Techniques for Characterization of Devices

Over the recent years a number of measurement methods have been developed in order to properly characterize the impact of defects on the device behavior. Most of the methods aim at applying a high stress bias for a specific period of time, and afterwards the state of the device is evaluated considering various ways. For instance stress-IV measurements, where IDVG sweeps are measured after a stress cycle has elapsed [17] have been used, but also hysteresis measurements [36,37], CV measurements [38,39], DLTS measurements [40,41,42,43,44] and on-the-fly methods [45,46,47] have been applied for assessment of the impact of charge trapping on the device performance. A common observation of the many measurement techniques used is that the $\Delta V_{\mathrm{th}}$ is observed to recover very fast, as soon as the stress bias is released [26,48,49,50]. To circumvent this limitation ultra-fast measurement setups have been developed [51,52]. With these methods short measurement delays of a few tens of nanoseconds can be achieved, whereas conventional tools exhibit delays in the hundreds of microseconds regime. The ultra-fast methods clearly reveal a significantly larger $\Delta V_{\mathrm{th}}$ [52] at nanoseconds delays. However, a considerable disadvantage of the high-speed methods is a typically high measurement noise of more than 10 mV in $\Delta V_{\mathrm{th}}$, as the signal-noise-ratio decreases at higher signal bandwidth. Thus the ultra-fast methods do not allow to resolve single charge transitions which are typically in the order of a few microvolt up to 10–15 mV [53,54]. However, a high measurement resolution is inevitable to study the physical mechanism of charge trapping, which has to be performed at the single defect level.

**Figure 2:**
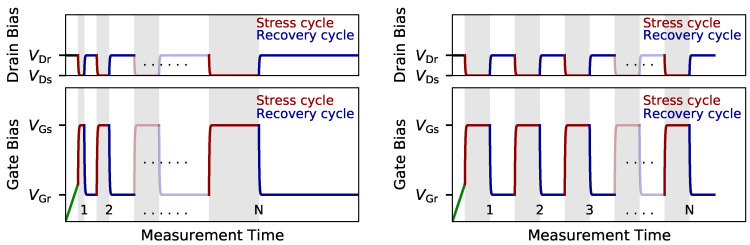
Schematic of the bias signals which are typically used for the measure-stress-measure (MSM) scheme which is applied for (**left**) the characterization of large-area devices, and (**right**) for defect-spectroscopy in nanoscale devices. An IDVG sweep is measured within a narrow gate bias range before the first stress cycle is applied. While the stress and recovery times are successively increased after each cycle for the characterization of large-area devices, *N* repetitions of the same sequence are performed when single-defects in nanoscale devices are studied.

To perform single defect spectroscopy MSM sequences are typically used. Patterns for MSM characterization of charge trapping in large-area devices and miniaturized transistors are shown in Figure 2, and rely on repeatedly applying stress and recovery cycles.

Before the first stress cycle is applied, an IDVG sweep within a narrow gate bias range is typically performed. As mentioned before, the IDVG characteristics serves for the calculation of the $\Delta V_{\mathrm{th}}$ from the recorded drain-source current in a post-processing step. The narrow bias range of the voltage sweep is important in order to preserve the pristine state of the device, as a gate voltage sweep over a too wide bias range can already cause considerable degradation of the device characteristics. If large-area devices are characterized the stress and recovery time of the subsequent measurement cycles are continuously increased for each cycle. By doing so the number of traps which can contribute to the drift of the threshold voltage $\Delta V_{\mathrm{th}}$ successively increase. It has to be noted that, in order to accurately explain the so measured temporal behavior of $\Delta V_{\mathrm{th}}$ the entire measurement sequence has to be simulated [55], as the $\Delta V_{\mathrm{th}}$ also shows a considerable permanent degradation, that is, the $\Delta V_{\mathrm{th}}$ does not vanish at the end of each recovery trace, and otherwise the permanent part would not be described by the simulations. In contrast to MSM sequences with increasing stress/recovery times applied for the characterization of large-area devices, the a fixed timing is used for stress/recovery cycles when TDDS measurements employing scaled transistors are performed. The main idea is that the defects which emit their charge during the recovery cycle get charged in the next stress cycle again and so on. In this way statistical information on charge capture and emission of defects can be collected and evaluated, which will be discussed in Section 4 in more detail.

**Figure 3:**
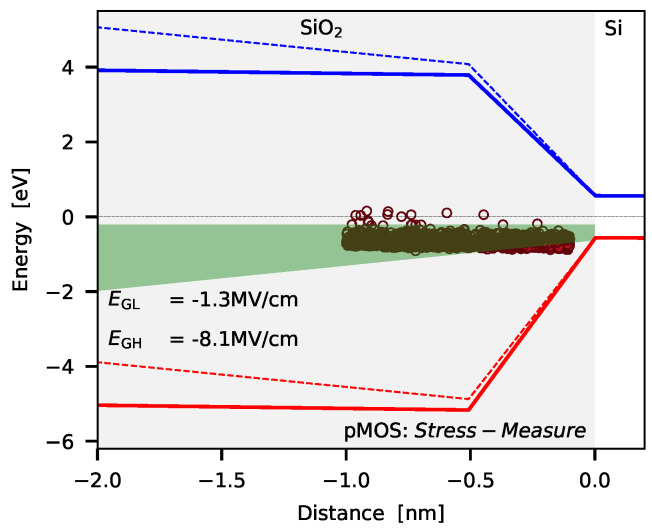
The band-diagram of a pMOS transistor is shown with a possible trap band of defects being responsible for the drift of the threshold voltage when negative BTI (NBTI) is considered. Also shown is the active energy region (AER, green area) for charge trapping which defines the energetic area of the defects which can contribute to the measurement signal at given bias conditions. The transition region shown in the band-diagram between the Si bulk material and the insulator which is in accordance with ab-initio calculations [56,57,58]. Quite recently, BTI in various technologies has been successfully explain using the modified band-structure [55].

An important criterion when applying MSM measurements is the energetic and spatial distribution of the traps which can contribute to the measurement signal. One condition for charge trapping concerns the timing of the MSM sequence and the charge capture and charge emission time of the defects at the respective bias condition and device temperature. The second boundary condition for charge trapping is defined by the stress and recovery bias used for the experiment. These biases determine the so called active energy region (AER) for charge trapping which is shown in Figure 3 for the NBTI/pMOS case.

In principle, the defects which exhibit a trap level below the Fermi level of the channel can become charged, and the defects with a trap level above the Fermi level remain neutral. Thus the key prerequisite of a defect to change its charge state during an MSM cycle is that its trap level is shifted below the Fermi level of the channel during the stress phase, but lies above the same during the recovery phase. The green area shown in Figure 3 is the energetic region where this condition is fulfilled, and thus marks the energetic area for defects which can affect the device behavior. Also shown is the hole trap band, which has been extracted for planar pMOS devices employing MSM measurements [55]. For this the reliability simulator Comphy has been used, which relies on the non-radiative multiphonon (NMP) defect model [34]. Next, the main properties of BTI are briefly discussed and afterwards defect models used to explain charge trapping are outlined.

## 3. Patterns of Bias Temperature Instabilities

The impact of BTI on the device characteristics is typically expressed in terms of an equivalent shift of the device threshold voltage $\Delta V_{\mathrm{th}}$, which can be calculated from the drain-source current behavior using an IDVG characteristics [59]. In general, the impact of BTI on devices can be classified into positive BTI (PBTI), where a positive gate bias is applied at the gate terminal of the MOS transistor during stress, and negative BTI (NBTI), which is referred to when a negative stress bias is used [60]. In the literature mostly the NBTI/pMOS case is considered as in this case the $\Delta V_{\mathrm{th}}$ appears more pronounced compared to the PBTI/nMOS case. The main reason lies in the about ten times higher trap density present in pMOS devices compared to their nMOS counterparts [61], which makes the assessment of the later with generalized measurement difficult. It has to be mentioned at this point, that recently a custom-designed defect probing instrument has been proposed and used to characterized NBTI and PBTI at $\Delta V_{\mathrm{th}}$ resolution of a few tens of micro-volts [53]. Despite the challenges for instrumentation, the experiments are typically conducted at accelerated stress conditions, that is, significantly larger biases and temperatures, as used for nominal device operation. The idea is to accelerate device degradation and recovery and to calibrate the models to the corresponding measurement data. Afterwards, the calibrated tools are used to estimate the impact of BTI on the device performance at normal operating conditions. This procedure, however, requires accurate physical models in order to ensure high quality of the extrapolations. Thus suitable models have to be able to explain the different patterns of BTI at various stress and recovery bias conditions and also capture the temperature activation of charge trapping. The most basic properties of BTI are briefly summarized next.

### 3.1. Temperature Dependence of Charge Emission Times

**Figure 4:**
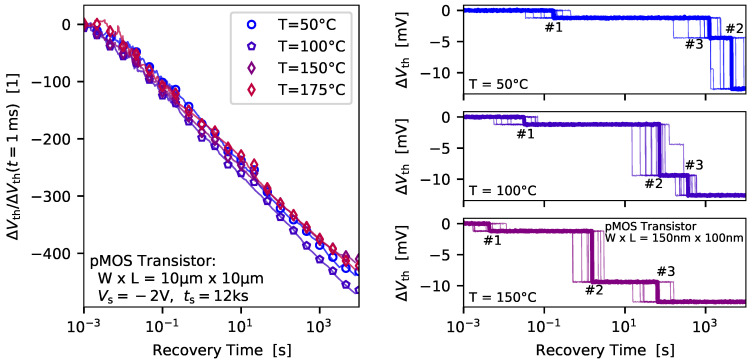
The temporal recovery behavior of a large-area and a nanoscale pMOS transistor is shown at different device temperatures. (**left**) Quite interestingly, the recovery traces of a large-area transistor can show only a very week temperature dependence when normalized to a certain reference value. (**right**) However, single-defect investigations clearly reveal a significant temperature dependence of charge trapping. Also shown here for the nanoscale device are the single recovery traces recorded at the same bias conditions, stress time and device temperature, which are used to determine the average emission time of the visible defects.

Several recovery traces recorded at the same stress and biases conditions but at different temperatures are shown in Figure 4 (left) for a large-area transistor. The traces have been normalized to $\Delta V_{\mathrm{th}}\left(t_{r}=1\;ms\right)$. As can be seen, a similar trend for the recovery behavior of the $\Delta V_{\mathrm{th}}$ can be observed at different temperatures. This indicates, that only a weak temperature dependence of charge trapping can be extracted from these measurements, which is an important parameter for developing of charge trapping models. But a significant change of the emission time can be observed when the average emission time of defects in nanoscale devices is evaluated, see Figure 4 (right). With increasing device temperature the defects move towards shorter emission times, clearly indicating a considerable temperature activation of the charge trapping kinetics. Although both cases rely on the same physical mechanisms, significant differences in thermal activation can be observed. This underlines once more the importance of investigating the behavior of individual defects in detail and taking this into account in the models.

### 3.2. Bias Dependence of Charge Trapping

**Figure 5:**
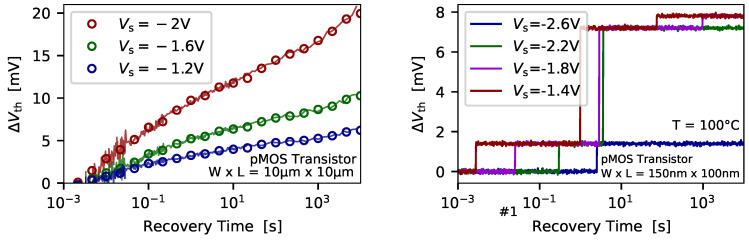
The temporal behavior of the drift of the threshold voltage is shown for pMOS transistors with two different geometries. (**left**) For large-area devices a continuous drift of the $V_{\mathrm{th}}$ can be observed. As more defects become charged at higher stress biases the recorded $\Delta V_{\mathrm{th}}$ increases too. (**right**) In nanoscale devices the number of single charge transitions, that is, the number of discrete steps in the $\Delta V_{\mathrm{th}}$, increase with higher stress bias. Also the average charge capture time move toward smaller values at higher stress biases.

**Figure 6:**
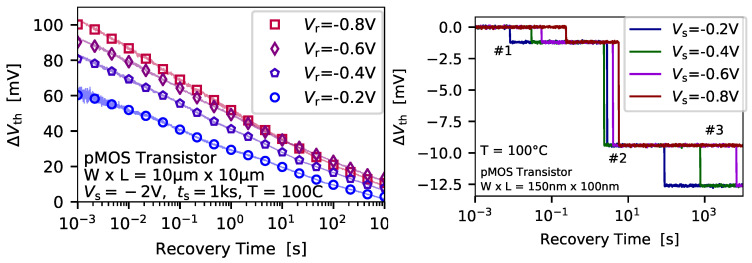
The recovery of the threshold voltage for pMOS transistors is shown from the perspective of a (**left**) large-area transistors and (**right**) a nanoscale transistor. In case of large-area devices the $\Delta V_{\mathrm{th}}$ shift which recovers appears to be seemingly lower at lower recover bias. However, the main reason for this observation is that the trap level of most of the defects is shifted far above the Fermi level of the channel, compared to the case for larger recovery biases, which leads to small charge emission times below the measurement delay. Thus, a significant bias dependence of the overall device recovery can be observed. The recovery behavior of defects from a nanoscale device exhibit emission times which can be either change with recovery bias (defects #1 and #3), or can be independent of the selected recovery bias (defect #2). Also remarkable is that defects can become shifted outside measurement window when the recover bias becomes too large.

The bias dependence of charge trapping is shown in Figure 5 for different stress biases and the impact of the recovery bias on the measured $\Delta V_{\mathrm{th}}$ is visible in Figure 6 for both a typical large-area and a representative miniaturized device. From Figure 5 (left) it becomes evident that at higher stress bias a larger shift of the threshold voltage $\Delta V_{\mathrm{th}}$ can be recorded. This observation can be explained by an increase of the AER at higher stress bias, and thus more defects are shifted above the Fermi level of the channel during the stress phase, and as a consequence more defects can become charged. In additions to the more defects shifted below the Fermi level, the energy difference between the trap level and the Fermi level increase at higher stress bias. Thus, the larger this energy gap gets the shorter the charge capture times become. This trend can be clearly observed when the charge capture events of defects in nanoscale devices are evaluated, see Figure 5 (right). Another similarity between large-area and nanoscale devices is the increasing number of defects which become charged when the stress bias is increased. Quite interestingly, while for large-area devices charge capture and charge emission are observed to be bias dependent, compare Figure 5 (left) and Figure 6 (left), a notable number of defects in nanoscale device exhibit bias independent charge emission times. This behavior can be observed for defect #2 from Figure 6 (right), whereas the two other defects #1 and #3 emit their charge at shorter emission times at lower recovery bias. In general, the bias independent emission time behavior is associated with so called *fixed traps*, whereas defects exhibiting a bias dependent emission time are typically referred to as *switching traps*. Thus, providing an accurate model to explain the bias dependence of BTI is pretty challenging, as the field dependence of individual defects is observed to be on one hand negligible and on the other hand very strong. In order to explore a more detailed picture of the many peculiarities of the charge trapping kinetics of defects in miniaturized devices the recent findings employing the TDDS are discussed next.

## 4. Time-Dependent Defect Spectroscopy of Metal-Oxide-Semiconductor (MOS) Transistors

Most of the characterization techniques proposed to investigate defect distributions and densities at various bias and temperature conditions employing large-area devices. One prominent example is the so called deep level transient spectroscopy (DLTS) [40] which has been adopted to extract the interface state density of MOS transistors [62]. In DLTS the interface traps can get charged by majorities when an accumulation pulse is applied. When the bias is switched to deep inversion, the traps emit their charge which can be observed as a temporal change in the device capacitance.

The time-dependent defect spectroscopy (TDDS) makes use of the principle of DLTS, applies it to miniaturized devices and augments it by a statistical analysis. The main prerequisite of TDDS is that the devices are small enough to reveal charge transition events as discrete steps of measurable size in the device current. According to recent reports the step height of the defects is proportional to the effective gate area, that is, $\eta =A\eta_{0}$ [61,63,64,65,66]. In contrast, the number of traps significantly decreases with the device geometry, that is, $N_{T}=N_{T}0/A$ [66]. Quite remarkable, in most recent technologies less than one trap per device can be present, however, its impact can evolve so pronounced that a charge transition can lead to a serious change of the device characteristics. Thus the proper operation of a single device can be solely determined by only one defect. Furthermore, the step heights produced by the individual defects which have been observed in single-defect investigations are widely distributed, ranging from several tens of micro-volts up to more than 30 mV and even higher depending on the device geometry [53]. To approximate their distribution an exponential distribution can be used [53,67]. The detection limit of the steps is basically given by the limited drain/source current measurement resolution of the instruments used. Note that for TDDS often custom-designed circuits are used enabling highest measurement resolution and performance [53].

The procedure to extract their charge transition kinetics, that is, their respective charge capture and emission times, as well as their steps heights will be discussed next in great detail.

### 4.1. Extraction of Charge Emission Time

**Figure 7:**
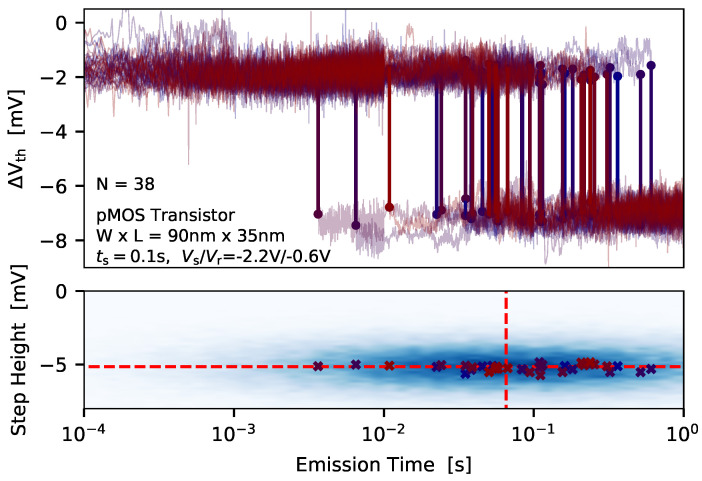
To extract the average charge capture time of a defect at a certain recovery bias the MSM sequence from Figure 2 (right) is applied, and *N* cycles at the same biases, temperature and stress/recovery times are measured. (**top**) The discrete steps in each recovery trace are extracted and binned into a (**bottom**) 2D histogram, which is called spectral map. In case that a number of emission events from a certain defect is available, a cluster is formed in the spectral map. Each of the clusters can be considered the finger print of a defect. The dashed lines in the spectral map indicate the average emission time, and the average step height, of the defect.

To extract the average charge emission time at a certain gate bias the measure-stress-measure (MSM) scheme from Figure 2 (right) is applied. As already mentioned, during the stress phase a number of defects is energetically shifted below the Fermi level of the channel can become charged. After a certain stress time has elapsed the gate bias is switched to a recovery bias, and the current through the device is recorded, and afterwards mapped to an equivalent $\Delta V_{\mathrm{th}}$ which is shown in Figure 7 (top).

**Figure 8:**
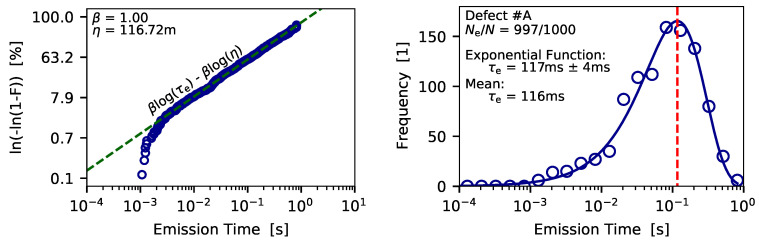
(**left**) The Weibull percentile function of the emission times indicates that the emission times follow an exponential distribution. (**right**) By binning the emission times into a histogram and applying the exponential distribution function the average emission time of the defect can be calculated. An almost equal average emission time is obtained when the mean value of transition times is calculated.

If the device is small enough discrete steps, which correspond to charge emission events of defects, can be observed. Afterwards, a step detection algorithm is applied to the measurement data in order to extract the charge transition events [68,69], which are then binned into a 2D histogram called spectral map, see Figure 7 (bottom). As can be seen, the charge emission transitions form a cluster in the spectral map, which is considered the fingerprint of the defect. The average step height of the defects can be considered to follow a normal distribution due to the measurement noise. To check for the distribution of the single emission time instances the bull percentile function can be analyzed [70,71], see Figure 8 (left).

For this the probability estimator [72]

(1) $$F=\frac{i-0.3}{N_{e}+0.4},$$

with *i* being the rank of the data point in the emission time series sorted in ascending order, and $N_{e}$ is the total number of emission events which are assigned to a certain defect. In case of β=1, as can be seen for the log-linear function in Figure 8 (left), the Weibull distribution function transfers to an exponential distribution function

(2) fWB(x)=λβ(λx)β−1e−(λx)β→XXβ=1XXfEXP(x)=λe−λx,

with $\lambda =1/\tau_{e}$. Alternatively, the exponential distribution of the charge emission events also becomes evident when the emission time points are binned into a histogram, see Figure 8 (right). It has to be noted that the quality of the histogram depends on the number of data points available and on the number of chosen bins. A more direct approach to calculate the average charge emission time is to calculate the mean value of the considered emission events

(3) $$\tau_{e}=\frac{1}{N_{e}}\sum_{i=0}^{N_{e}-1}\tau_{e,i}.$$

As noted in Figure 8 (right), by doing so the average emission time calculated lies well within the uncertainty of the estimation using the exponential distribution function. In a next step the charge capture of the defects has be extracted which will be discussed.

### 4.2. Extraction of Charge Capture Time

**Figure 9:**
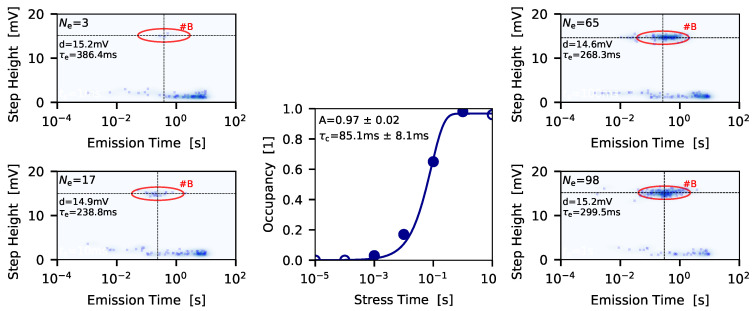
To extract the charge capture time a series of spectral maps (**left** and **right** images) for sequentially increasing stress times is recorded. From each spectral map the occupancy, that is, the ratio between the number of emission events of a certain defect and the number of traces measured, can be extracted. The occupancy follows an exponential behavior (**middle**) enabling to extract the charge capture time at a selected stress bias and device temperature.

In contrast to the direct extraction of the charge emission time from the recovery traces, the charge capture time cannot be determined directly, but can be extracted employing an indirect approach. For charge capture it can be assumed that the longer the stress time is the larger the probability of a defect to get charged becomes, when the same stress bias is considered. Thus, the expectation value of the occupancy, that is, the ratio between the number of recovery traces in which an emission event of the corresponding defect can be observed $N_{e}$ and the total number of traces measured $N_{N}$, follows

(4) $$O\left(t_{s}\right)=A\left(1-e^{-\frac{t_{s}}{\tau_{c}}}\right),$$

with *A* the occupancy and $\tau_{c}$ the charge capture time. The correlation between different stress times and the occupancy function is shown in Figure 9 (middle). As can be seen from the corresponding spectral maps for defect #B, with increasing stress time the respective cluster becomes brighter, that is, the occupancy $O=N_{e}/N_{N}$ increases. After the values for the occupancy have been extracted at a number of different stress times, the charge capture time can be estimated by applying Equation (4).

To determine the charge emission times over a wider bias range, the extraction method has to be performed for various stress biases. The upper limit for the stress bias is the breakdown voltage of the oxide, and the lower limit is given by the trap level of the defect, as this has to be shifted below the Fermi level of the channel during the stress phase. It has to be noted that, especially for defects with large capture time, the extraction scheme can be very time consuming. In order to extend the the measurement window for slow defects, the measurements can be performed at higher device temperatures, which can significantly elevate the extraction of the charge transition times at low stress biases.

The next steps is to provide an explanation for the extracted charge trapping kinetics of the defect. One promising approach relies on the non-radiative multiphonon theory, and will be amongst others discussed in the following.

## 5. Modeling of Charge Trapping

Most models developed to explain BTI aim at the reproduction of the temporal behavior of the $\Delta V_{\mathrm{th}}$ at different stress and recovery biases and at different device temperatures. The measured $\Delta V_{\mathrm{th}}$ typically shows a recoverable component, that is, the part of $\Delta V_{\mathrm{th}}$ which can be observed during the recovery cycle, and a permanent component, that is, the fraction of $\Delta V_{\mathrm{th}}$ which remains at the end of the respective trace. Thus, a suitable model necessarily has to be able to explain both contributions to the measured threshold voltage shift precisely.

A straight-forward approach to explain the experimental data is to use empirical models. However, such models typically aim at describing the data by simple mathematical formulas, but omit the detailed physical mechanism behind the phenomena. In the context of device physics experimental data can often be modeled using a power law or exponential-like functions [73,74]. Although empirical models can be used for comparing different technologies, they have to be treated with care as they do not provide a physics based explanation for the observations. Thus extrapolations of the data, for instance to estimate the device lifetime, may not be very accurate. Another disadvantage of empirical models is that they have been developed to explain a continuous trend in device threshold degradation and recovery, and are not designed to explain the discrete charge trapping behavior of scaled devices. To describe such a device behavior a stochastic charge trapping model is required rather than an approximation by a simple power law.

Attempts for the description of charge trapping have been based on the assumption that charge capture an emission can be explained by an elastic tunneling process [75,76,77]. During an elastic tunneling process a charge carrier can transit from a reservoir, that is, the device channel, to a respective defect site and get trapped without changing its energy. In this case, the charge transition rates are found to be proportional to the trap depth, $\tau \propto \exp-x/x_{0}$, which introduced difficulties when describing the large charge transition times for miniaturized devices which exhibit thin oxides [78,79]. Another limitation of elastic tunneling models is that the tunneling process is almost temperature independent, which cannot account for the considerable temperature dependence of charge trapping, see Figure 4 (right). As a consequence, models which assume elastic tunneling may not provide an accurate description of charge trapping considering BTI.

**Figure 10:**
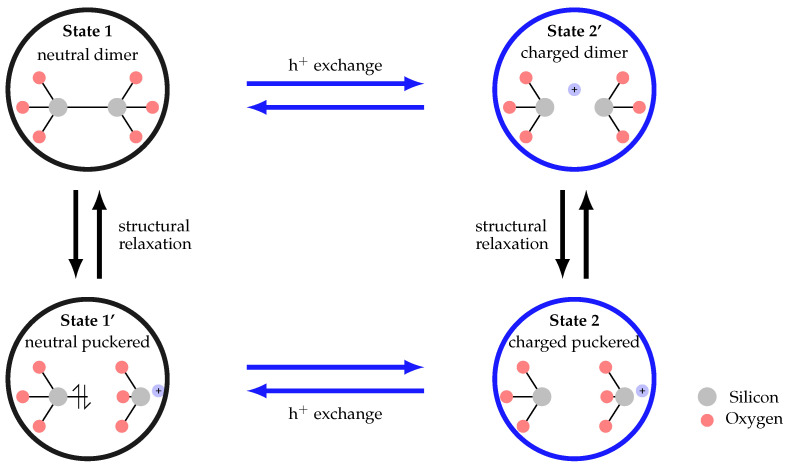
The non-radiative multiphonon (NMP) defect model has been proposed to explain the charge trapping kinetics of single defects. The model considers four defect states, two neutral defect states 1 and 1’ and two charged defect states 2’. The prime states are considered the meta-stable states of the system whereas the other states are the stable states. Either by exchanging a charge carrier or by structural relaxation the defect can charge its current state within in the NMP model. For a certain defect candidate, here shown for the E’ center, a certain atomic configuration of a defect can be assigned to one of the states of the defect model.

A very promising approach to model BTI was initially proposed in Reference [78] and has been refined in References [34,80]. The model is based on the concept of charge trapping which has been introduced to describe the stochastic nature of noise signals, that is, RTN and 1/f noise [81,82] and relies on hole trapping at defect sites which are located in the oxide supported by a multiphonon emission (MPE) process [75,83]. With MPE processes considerably larger charge capture and emission times can be achieved, which makes the model more suitable for BTI [84]. In the initial approach the HDL model has been used to explain charge trapping of switching oxide traps [85]. One characteristics of switching oxide traps is that their charge capture and emission time are bias dependent. Such a behavior can be described by three-state defect model. Later a notable number of single defect studies revealed that defects can also exhibit bias independent charge emission times. Such a behavior is referred to as fixed oxide traps [80]. Such a behavior can be described by the introduction of an additional defect state to the HDL model, leading to the four-state defect model shown in Figure 10.

The four-state NMP model consists of two stable states (1 and 2) and two metastable states (1’ and 2’). In the model the transitions between the defect states are either described by an NMP process for the transitions where a charge exchange takes place, that is, $1\to 2^{\prime }$ or $2\to 1^{\prime }$, or by a thermal barrier, that is, $1\to 1^{\prime }$ or $2\to 2^{\prime }$, where the defect undergoes a structural relaxation but does not change its charge state. A significant difference between both barriers is that the charge transfer reaction leads to bias dependent transition times, while the thermal barriers results in bias independent transition times. In order to ensure the physical accuracy of the model an atomic configuration of a certain defect candidate can be assigned to each state of the model. In Figure 10 the atomic configurations of the so called E’ center, which have been calculated using *ab-initio* methods, are shown [86]. This defect class has been proposed as hole trap candidate in pMOS transistors [87,88]. Further trap candidates are defects involving hydrogen, namely defects in the hydrogen bridge configuration [89,90] or hydroxyl $E^{\prime }$ centers [91]. The elongated oxygen bond has been proposed as suitable electron trap candidate for charge trapping in nMOS devices [92].

In the final section of this paper the different charge trapping behavior of defects which have been observed from single defect investigations and the corresponding configuration of the defect model to explain the trap behavior is discussed.

## 6. Results

In the following, results from single defect studies performed on nanoscale devices are discussed in detail. The shown charge trapping kinetics has been extracted either by applying TDDS, or from RTN measurements, and is modeled considering the four-state defect model. It can be observed that the model nicely explains the experimental data. In addition to the charge trapping kinetics, the impact of the defects on the device behavior is also an important parameter for device reliability assessment. This can be analyzed by calculating distribution function of step heights of the single charge transition events, which is subject of the second part of this section.

### 6.1. Charge Trapping Kinetics of Single Defects

Extensive studies employing the previously mentioned TDDS have been carried out using utilizing pMOS and nMOS transistors. These investigations reveled many peculiarities visible in the charge trapping kinetics of the defects, which all have to be covered by a uniform model. It has been observed that the charge emission times of traps can be either (i) bias-dependent, which is typically referred to as *switching trap*, or (ii) bias-independent, a behavior which is assigned to so called *fixed oxide traps*. In both cases strong bias dependent charge capture times are observed. Another remarkable observation is that (iii) defects can show a volatile behavior [93]. More detailed, a small number of defects have been observed to vanish from the spectral map and some of them reappeared in the spectral maps at a later time point. It has to be noted that volatile defects have been observed in nMOS and pMOS devices using SiON and high-k gate stacks and are thus not limited to any particular technologies. As the phenomenon is stochastic, it is very difficult study it systematically. However, these defects will an essential clue on the chemical nature of oxide traps.

**Figure 11:**
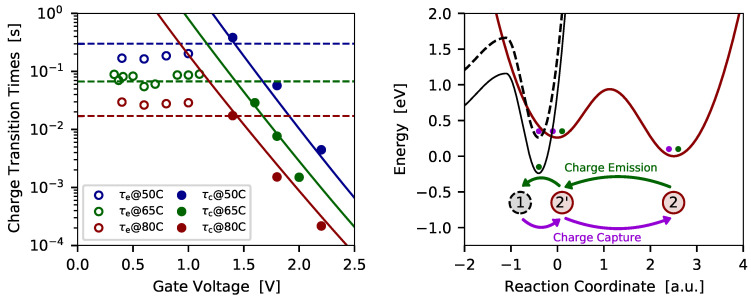
(**left**) The charge trapping kinetics for a *fixed trap* is shown at different temperatures (symbols ... measurement data, lines ... simulations). As can be seen, the fixed trap shows bias dependent charge capture times, but bias independent charge emission times. (**right**) To explain this behavior three states of the defect model are used and the pathways for charge capture an emission are shown together with the corresponding approximation for the potential energy surfaces.

**Figure 12:**
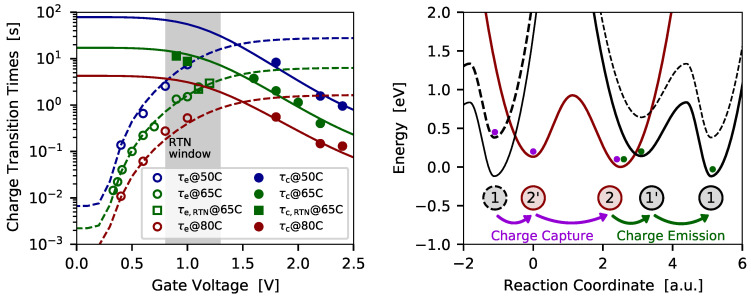
(**left**) The charge transition times of a *switching trap* shows bias dependent charge capture and charge emission times (symbols ... measurement data, lines ... simulations). (**right**) To explain the bias and temperature dependence the four state defect model is used and the corresponding approximation for the potential energy surfaces (PES) is shown. This kind of defect requires four defect states in order to properly capture the trapping behavior.

In Figure 11 (left) and Figure 12 (left) the charge trapping kinetics of two defects which have been extracted from SiON pMOS transistors is shown.

The defect presented in Figure 11 (left) shows a fixed trap characteristic with bias-independent charge emission times, but bias dependent charge capture times. The corresponding configuration coordinate diagram with the potential energy surfaces (PESs) used to describe the charge transitions is given in Figure 11 (right). As already mentioned, the energy of the atomic configuration of the different defect states of the NMP model is calculated using density functional theory. The transitions from one defect state to another are then approximated by a harmonic oscillator, which is represented by the PESs. The PESs either describe the situation of a neutral defect where the carrier is in its reservoir, or describe the situation where a carrier is trapped at a defect. A transition between the two states, that is, a charger transfer reaction, can occur when a carrier surpasses the energy barrier between two states. To account for the bias dependence the relative position of the PESs is shifted according to the change of the trap level when a gate bias is applied at the device. In case of a fixed trap, the transition barrier between the states 1 and 2’ becomes relatively small when a gate bias is applied, see dashed PES in Figure 11 (right). The system can further overcome the thermal barrier between the states 2’ and 2, and finally transit to the stable charge state 2. In summary, the charge transition proceeds via the pathway $1\to 2^{\prime }\to 2$. The switching trap from Figure 12 follows the same pathway when a charge capture event occurs. However, the charge emission behaviors different for both cases. In case of the fixed trap, the thermal barrier between the states 2 and 2’ determines the charge emission process, while the barrier between the states 2’ and 1 is very small, see solid PES in Figure 11. Thus, the charge emission follows the pathway $2\to 2^{\prime }\to 1$. In contrast, charge emission for the switching trap proceeds via the pathway $2\to 1^{\prime }\to 1$. Here the barrier between the states 2 and 1’ (solid PES in Figure 12) determines the charge emission time. It has to be noted that the charge transition processes, meaning the transitions between different charge states of a defect, can be observed in the measurements as discrete steps in the current. The thermal barriers are given by the overall charge trapping dynamics, but transitions via these barriers are not directly visible in the measurement data.

Once the defect model is calibrated to a number of defects the parameters can be extended to explain BTI in large-area devices. For this the trap levels and energy barriers are considered distributed, which enables to calculate a number of defects with different configuration of their PESs. Finally, the superposition of an large ensemble of defects allow explanation of BTI in large-area devices [94]. Based on this accurate lifetime estimations can be made. Quite recently, the two-state defect model has been implemented into a 1D reliability simulator Comphy [55] and successfully applied to explain BTI in various technologies. Lately it has also been demonstrated that the defect model in combination with TCAD simulations can nicely explain charge trapping in SiC transistors, where a good agreement between the extracted trap parameters and results from DFT calculations has been observed [11]. Furthermore, it has been demonstrated that empirical models typically omit effects like saturation of the $\Delta V_{\mathrm{th}}$ with increasing stress time, but rather predict indefinitely large $\Delta V_{\mathrm{th}}$ when the stress time becomes very large. However, such extrapolations are rather un-physical and pessimistic, thus a physics based approach for explaining charge trapping, like the NMP defect model in combination with TCAD simulations, considering the charge trapping kinetics of single defects to explain charge trapping is preferred.

### 6.2. Distribution of Step Heights of Single Defects

**Figure 13:**
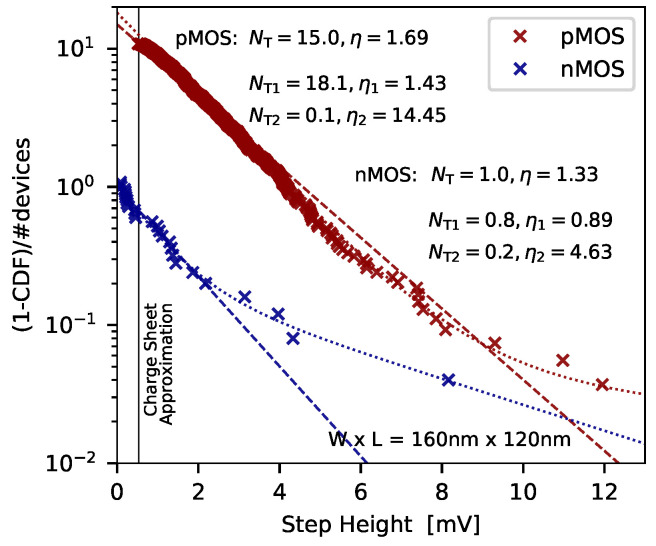
The complementary cumulative distribution function (CDF) is shown for nMOS and pMOS SiON transistors of equal active gate area. The insulator thickness is $t_{\mathrm{ox}}$ = 2.2 nm for all devices. As can be seen, the complementary CDF reveals two branches for both kinds of transistors. Such a behavior can be well described by Equation (8) (dashed lines consider uni-modal exponential distribution, dotted lines consider bi-modal exponential distribution). Additionally, the maximum step height calculated considering the CSA is also shown (solid black line). As can be clearly seen, the CSA significantly underestimates the effective impact of the single defects on the overall shift of device threshold voltage. Furthermore, it can be seen the number of active traps seems to be higher in pMOS devices compared to the nMOS counterparts.

To estimate the impact of a single defect on the device behavior the charge sheet approximation (CSA), which assumes that the oxide charge is spread over the insulator according to [75]

(5) $$\Delta V_{\mathrm{th}}=-\frac{q}{\epsilon_{0}\epsilon_{r}WL}t_{\mathrm{ox}}\left(1-\frac{x_{T}}{t_{\mathrm{ox}}}\right),$$

with the elementary charge *q*, the dielectric constants $\epsilon_{0}$ and $\epsilon_{r}$, the oxide thickness $t_{\mathrm{ox}}$ and the position of the trap $x_{T}$, is typically used. By applying the CSA the trap density can be estimated from a given $\Delta V_{\mathrm{th}}$ [55,95]. However, considering the CSA typically leads to an overestimation of the trap density, as the real average impact of a defect on the overall $\Delta V_{\mathrm{th}}$ has been observed to be more pronounced, when measurements of different technologies are evaluated [25,49,96]. In order to determine the average impact of a single trap on the $\Delta V_{\mathrm{th}}$, the distribution function (CDF) of step heights has to be created and analyzed [25,96]. To extract the CDF stress-recovery measurements have to be performed employing a number of devices of the same technology. For each device one recovery trace is measured after the device has been stressed for typically 1 ks at oxide fields up to 10 MV/cm. Afterwards, the charge transitions of each trace are extracted and the CDF created, see Figure 13.

It can be seen, that the step heights are exponentially distributed and can be described by the respective probability distribution function (PDF)

(6) $$f\left(\Delta V_{\mathrm{th}}\right)=\frac{1}{\eta }e^{-\frac{\Delta V_{\mathrm{th}}}{\eta }}$$

with η the mean threshold voltage shift caused by a single charge transition event of a certain defect. From the PDF the cumulative distribution function (CDF) can now be calculated

(7) $$F\left(\Delta V_{\mathrm{th}}\right)=\int f\left(\Delta V_{\mathrm{th}}\right)d\Delta V_{\mathrm{th}}=1-e^{-\frac{\Delta V_{\mathrm{th}}}{\eta }}.$$

To study the distribution of the step heights the complementary CDF is used, and is evaluated normalized to the number of devices

(8) $$\frac{1-CDF}{\#devices}=\sum_{i}N_{i}e^{-\frac{\Delta V_{\mathrm{th}}}{\eta_{i}}},$$

with $N_{i}$ the average number of active defects per devices. The expression above already accounts for multi-modal behavior of the experimental complementary CDFs. Note, one advantage of the normalization of the complementary CDF is that the number of traps per device is directly accessible from the plots.

A remarkable observation here is that the distribution function of the step heights follow a bi-modal exponential distribution. Recent studies [63] suggest that the bi-modal exponential distribution is typical for devices employing high-k gate stacks, where one branch is attributed to charge transfer reactions between the channel and the high-k layer, and the second branch accounts for channel/${\mathrm{SiO}}_{2}$ trap interaction. However, it turned out that bi-modal exponential distributions can also be observed for devices with an SiON insulator [53,96]. In Reference [96] it has been suggested that the two branches of the complementary CDF measured from nMOS devices can be separated into gate/defect and channel/defect interactions.

Another important finding is that exponentially distributed amplitudes have also been found for RTN signals [97,98,99]. These findings strengthen the link between RTN and BTI [25,80]. Furthermore, the average contribution of a single trap to the threshold voltage shift η plays an important role in the context of device variability in deeply scaled devices [67,99,100,101].

## 7. Conclusions

The characterization and accurate modeling of the reliability of miniaturized transistors poses a major challenge for measurement instrumentation, defect modeling and device simulation. In order to explain the experimental observation empirical models are often used. However, such models typically omit certain observations, like saturation of the drift of the threshold voltage with increasing stress time. In order to provide a physical description of the measurement data the four-state defect model has been proposed, and is discussed here. The defect model is based on the charge trapping kinetics of single defects which can be observed in miniaturized devices. To extract the trapping behavior the time-dependent defect spectroscopy (TDDS) can be used. From recent TDDS studies it has been observed that defects exhibit bias dependent charge capture times, but certain defects exhibit bias-independent charge emission times while others show bias-dependent charge emission times. Both characteristics can be nicely explained by the defect model. To explain the behavior of large-area devices a number of defects with distributed trap levels and energy barriers for charge transitions have to be calculated, and their superposition enable to describe the devices’ behavior. These simulations can be further used to accurately extract the lifetime of the devices under various operating conditions. Finally, the distribution function of step heights is discussed, and it is shown that the typically use charge sheet approximation significantly underestimates the effective impact of a defect on the device behavior. This is especially important for circuit designers to ensure a high robustness of the applications against charge trapping.
